# Parasite-based malaria diagnosis: Are Health Systems in Uganda equipped enough to implement the policy?

**DOI:** 10.1186/1471-2458-12-695

**Published:** 2012-08-24

**Authors:** Daniel J Kyabayinze, Jane Achan, Damalie Nakanjako, Betty Mpeka, Henry Mawejje, Rukaaka Mugizi, Joan N Kalyango, Umberto D’Alessandro, Ambrose Talisuna, Van geertruyden Jean-Pierre

**Affiliations:** 1 Malaria Consortium, Upper Naguru East Road, P.O. Box 8045, Kampala, Uganda; 2 Foundation for Innovative New Diagnostics, Akii Bua Road Nakasero, Kampala, Uganda; 3 Makerere University College of Health Sciences, P.O. Box 7072, Kampala, Uganda; 4 Institute of Tropical Medicine, Antwerp, Belgium; 5 The Medical Research Council Unit, The Gambia; 6 Makerere University School of Public Health, P.O. Box 7072, Kampala, Uganda; 7 Malaria Public Health and Epidemiology Cluster, University of Oxford-KEMRI-Wellcome Trust Research Programme; 8 Unit International Health, ESOC Department, Faculty of Medicine, Antwerp University, Universiteiplein 1, BE-2610, Antwerpen, Belgium

## Abstract

**Background:**

Malaria case management is a key strategy for malaria control. Effective coverage of parasite-based malaria diagnosis (PMD) remains limited in malaria endemic countries. This study assessed the health system's capacity to absorb PMD at primary health care facilities in Uganda.

**Methods:**

In a cross sectional survey, using multi-stage cluster sampling, lower level health facilities (LLHF) in 11 districts in Uganda were assessed for 1) tools, 2) skills, 3) staff and infrastructure, and 4) structures, systems and roles necessary for the implementing of PMD.

**Results:**

Tools for PMD (microscopy and/or RDTs) were available at 30 (24%) of the 125 LLHF. All LLHF had patient registers and 15% had functional in-patient facilities. Three months’ long stock-out periods were reported for oral and parenteral quinine at 39% and 47% of LLHF respectively. Out of 131 health workers interviewed, 86 (66%) were nursing assistants; 56 (43%) had received on-job training on malaria case management and 47 (36%) had adequate knowledge in malaria case management. Overall, only 18% (131/730) Ministry of Health approved staff positions were filled by qualified personnel and 12% were recruited or transferred within six months preceding the survey. Of 186 patients that received referrals from LLHF, 130(70%) had received pre-referral anti-malarial drugs, none received pre-referral rectal artesunate and 35% had been referred due to poor response to antimalarial drugs.

**Conclusion:**

Primary health care facilities had inadequate human and infrastructural capacity to effectively implement universal parasite-based malaria diagnosis. The priority capacity building needs identified were: 1) recruitment and retention of qualified staff, 2) comprehensive training of health workers in fever management, 3) malaria diagnosis quality control systems and 4) strengthening of supply chain, stock management and referral systems.

## Background

Prompt and accurate diagnosis of malaria is part of effective disease management and a key strategy for malaria control in sub-Saharan Africa. Globally, 216 million cases and 655,000 deaths were reported in 2010. Up to 90% of all malaria deaths occurred in Africa [1]. Parasite-based malaria diagnosis (PMD) has been shown to improve quality of care, reduce drug consumption [2] and enable early treatment of non-malaria febrile illness [3,4]. In 2009, 33 of 43 malaria-endemic countries in the African region reported having adopted a policy of providing parasitological diagnosis for all age groups [1] including scale up of rapid diagnostic tests (RDTs) at lower level health facilities (LLHF) where microscopy is not feasible. However, equitable access and coverage of effective interventions remains limited due to the prevailing health care system challenges in resource-limited malaria-endemic settings in Africa [5].

Health care delivery systems are critical for individual countries’ responses to evidence-based, efficacious interventions to reduce malaria-attributable morbidity and mortality among the most vulnerable sub-populations. Limited institutional capacity is one of the common challenges in health systems yet it is a vital ingredient of effective services’ delivery. The building blocks of a functional health system include good health services, a performing workforce, functioning health information systems, essential medical supplies, a good health financing system as well as good leadership and governance [6]. A break in any one of the blocks can potentially limit the impact of an otherwise efficacious intervention [7]. With inadequate health care delivery system capacity, health spending on the right services and interventions may lead to minimal provision of the actual services [8]. For example, reports from Ghana and Tanzania showed that organizational, logistical and technical challenges affect implementation of PMD using RDTs [9,10]. Targeted capacity-building activities can translate into the expected efficiency if the weak links are pinpointed and addressed strategically [11]. Therefore, health planners and managers need to understand and address the bottlenecks to implementation and coverage of PMD among other health care services.

This study assessed the capacity of the health system to implement malaria diagnosis interventions at LLHF in Uganda. Assessment of the service delivery capacity needs was based on a four-tier hierarchy of capacity needs that include 1) tools, 2) skills, 3) staff and infrastructure, and 4) structure, systems and roles [11]. This was a sub-study within a wider assessment of management of severe malaria at LLHF in regions with medium and high malaria transmission settings in Uganda. The study identified priority health care system capacity building needs that require intervention in order to optimize PMD and malaria case management in general.

## Methods

### Study design and setting

This cross sectional survey assessed the health system capacity for malaria diagnostics at LLHF; health centre (HC) II and III that have a catchment of up to 10,000 and 20,000 people respectively. The larger LLHF (HC IIIs), led by a clinical officer (a clinical officer receives three years pre-service medical and surgical training), provide basic preventive and curative care, maternity care, initial pre-referral care, supervision to the HC II under their jurisdiction and laboratory diagnostic services including microscopy and RDTs. The smaller LLHF (HC IIs), led by a comprehensive nurse (a comprehensive nurse receives medical and midwifery pre-service training), have provision for RDTs although they do not have facilities for microscopy. This sub-study was conducted within a larger study to assess malaria case management practices at different levels of health care delivery within high malaria transmission (Eastern region) and low-medium malaria transmission (mid-Western region) regions in Uganda [12]. Multi-stage cluster sampling methods were used to select study health facilities. Of the 250 LLHF in the two regions, 125 (50%) were randomly selected for health system capacity assessment. This representative sample was deemed feasible and adequate to document capacity needs at LLHF. At the LLHF, all health workers found at the study facility were consecutively offered an interview based on study tools. Health facility specific questions were addressed to the head of the health facilities. The interviews were conducted by 5 teams (4 medical research assistants per team) working in parallel. The teams were trained for one week prior to the survey to ensure that interview questions were asked appropriately and responses consistently recorded. Most survey instruments were adapted from the WHO hospital care assessment tools (http://whqlibdoc.who.int/publications/2008/9789241596428_eng.pdf); see study tools as Additional file 1.

### Study measurements

**Figure 1:**
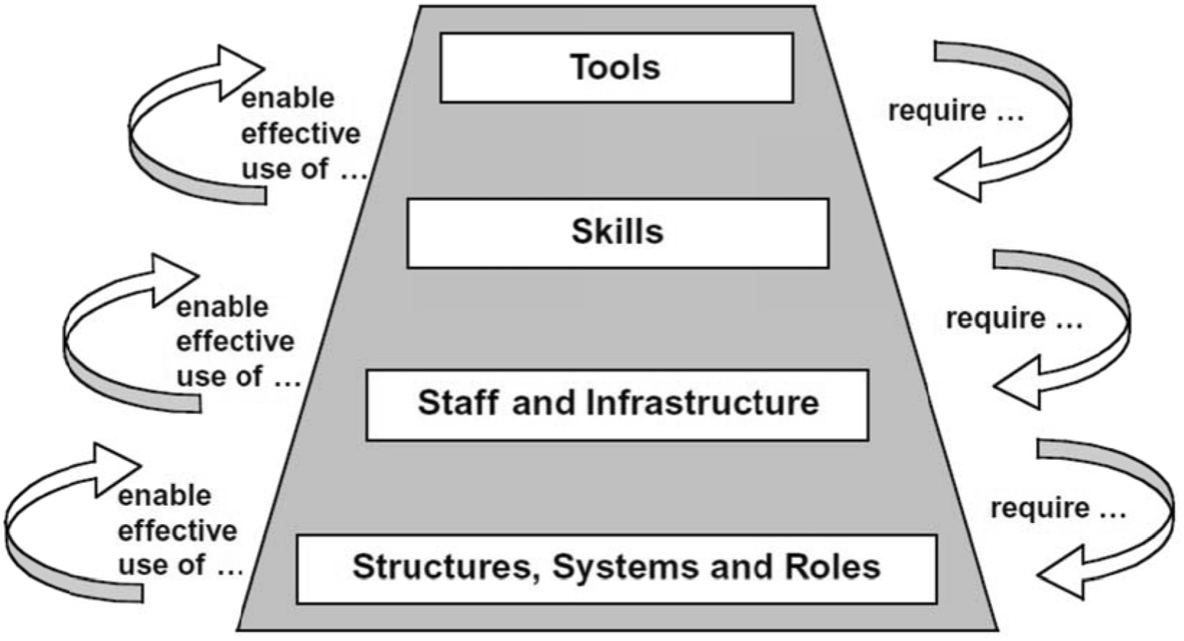
**Capacity pyramid.** Shows the capacity building hierarchy and the importance of the linkages between the various capacity building blocks.

The health system capacity assessment pyramid was used which considers a four-tier hierarchy of capacity needs; that is: 1) tools 2) skills 3) staff and infrastructure and 4) structure, systems and roles (see Figure 1). Using a pre-tested, pre-coded questionnaire, all health workers were interviewed about the available capacity and their perceived capacity building needs. Health facility assessment check lists were used by the research assistants to collect observed data.

The tools assessed included among others: availability of diagnostic kits, equipment, drugs and medical consumables as part of performance capacity for PMD. Routine availability and use of microscopy at LLHF were presented as proportions. However, this survey did not assess the quality of the tests done. The inventory system was assessed by the reported frequency and duration of stock-out period of anti-malarial drugs and other medical supplies. LLHF that reported at least one three-months-long stock out period were taken to have an inadequate inventory and stock management system.

Skills capacity was assessed by evaluation of the staffs’ individual knowledge, technical skills as well as training and motivation to perform their duties including malaria diagnostic tests. For knowledge assessment, health workers were asked about identification and management of severe malaria. Knowledge was graded inadequate if health workers mentioned only 1 out of 10 types of severe malaria, <4 out of 12 danger signs, only one cause of non-malaria febrile illness, could not identify parenteral quinine as the first-line treatment for severe malaria and did not mention two life saving practices for severe malaria. Previous training in integrated management of childhood illnesses was used as an indicator of skills training in management of febrile illnesses.

Staff and infrastructure were assessed by determining proportion of vacancies occupied by qualified health workers (as recommended by the Ministry of Health staffing norms as shown in Additional file 2). The proportion of filled positions was the number of employed qualified health staff over the expected number of staff. Given the 125 LLHF, (64 HCII and 61 HC III) the optimal professional health worker numbers expected is 730 (excludes nursing assistants). The health workers were asked about presence of job descriptions and use of standard operating procedures. Supervisory capacity was assessed by documenting frequency and source of health workers’ supervision. The methods and frequency of supervision were used to assess the regular reporting, monitoring and evaluation systems available. Facility capacity was assessed through asking about physical space for diagnostic activities, storage and mechanisms of safe disposal of infectious waste.

Structures, systems and roles capacity were assessed by determining availability of support services such as; supply chain management, coverage of microscopy quality control/assurance systems and patient referral systems. Health workers were interviewed about their drug ordering and stock management systems including questions about drug stock-out periods. In addition, we assessed the referral systems through which patients at LLHF can access care at larger hospitals. Review of clinical notes and patient/care-giver interviews were conducted on all patients with suspected malaria that were admitted at the district hospitals through referrals from LLHF to document the prevalent pre-referral and referral systems and practices. Data were collected on reasons for referral, use of referral notes, pre-referral medication and transport support. Patients were considered to have had adequate referral if they received all of; a) pre-referral medicine, b) a referral note and c) transport support [12] based on the Uganda Ministry of Health National Treatment guidelines, 2^nd^ Edition, of 2003. The Health Management Information System (HMIS) was assessed by availability of patient registers, completeness of registers, storage and utilization of records to inform drug stock management. Administrative and organizational capacity was assessed by documentation of available decision-making forums as evidenced by schedules and minutes from staff meetings. Management capacity was determined by cadres in leadership of the LLHF. LLHF leadership was deemed adequate if a HCII was headed by a comprehensive nurse and a HCIII by a clinical officer.

### Data analysis

Data were double entered in EPI-info software program version 6 and analyzed using STATA version 10.0 (StataCorp LP, College Station, TX, USA). Summary measures were presented as frequencies and proportions.

### Ethical approval

The study was conducted according to the principles of the declaration of Helsinki and the international guidelines of biomedical research involving human subjects (http://www.cioms.ch/frame_guidelines_nov_2002.htm). Ethical approval was granted by the Uganda National Council for Science and Technology (UNCST) which also acts as an ethics committee. Support for the study was acquired from LLHF leader and each participant provided verbal informed consent to participate since this was considered appropriate for a routine audit of the health services.

## Results

### Health facility characteristics

Between June and August 2009, a cross sectional assessment of health service delivery capacity was conducted at 125 health centers in Eastern and mid-Western Uganda. Of these, 110 (88%) were government owned facilities, 64(51%) were level II health centres (HC II), and 61 (49%) were level III health centres (HC III) (Table 1).

**Table 1:** Health service delivery capacity needs at 125 low level health facilities in Uganda between June and August 2009

**Assessed parameter**	**Characteristic**	**N(%)**
**Facility level**	HC II	64(51%)
	HC III	61(49%)
**Facility Ownership**	Government	110(88%)
	Faith Based	13(10%)
	Private	2(2%)
**Tools (Performance capacity)**		
Diagnostic Tools	Functional microscopy HCIII (n = 61)	18(30%)
	Available malaria RDTs(n = 125)	12(10%)
Antimalarial Drugs available for 3 months	Oral antimalarial drugs ACTs	70 (56%)
	Parental Quinine available (n = 91)	48(52%)
	Rectal Artesunate	1 (1%)
	Normal Saline (n = 9	33(38%)
	50% dextrose available	32(35%)
	Intra venous (IV) giving sets	38(42%)
	Syringes	51(56%)
Guidelines	Treatment guideline for malaria available	108(82%)
	Referral guidelines	None
**Staff and infrastructure‡**		
Work-load	Approved staff position filled with qualified staff	131(18%)
	In patient space for admission (HCIII n = 35)	9(25%)
Work-flow	Effective triage system in places (n = 111)	33(30%)
Support services	Waste management and infection control tools	Inadequate
Supply chain	Inventory system(stock cards, and consumption data)	None
Quality control	Laboratory external quality assurance (N = 18)	None
Support supervision	Supervision from Malaria focal person 6 months	12(10%)
**Information system**	Available Out-Patient (OPD) registers	125(100%)
	Up to date registers available	120(97%)
	Complete records with vital mortality information	25(20%)
	Computerised data systems	None
**Systems and Role Capacities**		
	Hold regular monthly meetings	47(56%)
	Health centre III led by Clinical Officers (n = 61)	9(15%)
	Health Centre II led by Comprehensive Nurse (n = 64)	36(56%)

### Tools

#### Performance capacity

PMD was available in 30/125 (24%) of the HC; 29% had functional microscopy and 20% had only RDTs in use. Health facilities lacked a well coordinated inventory system to ensure non-interrupted supply of medicines and reagents. Three months’ long stock-out periods were reported for oral and parenteral quinine at 39% (49/125) and 47% of LLHF respectively. Continuous 3 months’ availability of oral artemisinin-based combination therapies (ACTs) for uncomplicated malaria and sulphadoxine–pyrimethamine (SP) for intermittent preventive treatment of malaria in pregnancy (IPTp) was available in 56% (70/125) and 59% (74/125) respectively. Rectal artesunate for pre-referral medication was only available in one health centre. Up to 35% of the LLFH had continuous supplies of medical consumables (38% had normal saline, 42% had IV giving sets, 35% had in stock ‘dextrose fifty percent’ and 56% had syringes) for the three months preceding the survey. Nine out of the 125 LLHF (7%) reported lack of parental quinine throughout the year of the study. Similarly, 20% of the facilities lacked thermometers, weighing scales and simple hand held glucometers. Treatment guidelines were available in 86% (108/125) of the health centres.

### Skills

#### Personnel capacity

A total of 131 health workers found at LLHF were interviewed. Of these, 86 (66%) were nursing aide/assistants (Table 2). Overall, 47 (36%) of the health workers were graded as sufficiently knowledgeable in identifying clinical symptoms and signs of severe malaria. Only 56 (43%) health workers mentioned two or more forms of severe malaria, 72 (55%) where able to list at least four danger signs of severe malaria, 109 (83%) reported two differential diagnosis of malaria. However, less than 40% could mention at least two very important practices for saving life in patient with severe malaria (55% mentioned identification of danger signs and 38% mentioned a short but thorough examination of patient). The common forms of severe malaria mentioned by health workers were convulsion or fits (77%) and cerebral malaria (71%). Up to 97% knew that quinine reported that drug of choice for treatment was parenteral quinine Sixty two (47%) of the health workers had ever received training on Integrated Management of Childhood Illnesses (IMCI). Sixty six (50%) health workers had received supervision on malaria case management within 6 months preceding the survey. Majority 44 (67%) of the supervision was conducted by personnel outside the LLHF.

**Table 2:** Training and professional skills of health workers at lower level facilities in Uganda between June and August 2009

**Assessment parameter**	**Characteristics**	**N = 131**
**Pre-service training**	Clinical officers	9(7%)
	Mid wife	8(6%)
	Nurse	28(21%)
	Nursing aid/assistant	86(66%)
**Personal capacity**		
Skills	Knowledge of severe malaria danger signs*	47(37%)
	Received IMCI training	62(50%)
	Supervision in the preceding 6 months	55(42%)
Motivation	Received Job descriptions and appointment letters (n=124)	78(63%)
**Referral practice**(N = 186)	Pre-referral antimalarial drugs given (n = 186)	130(70%)
	Referral clinical notes given to patients	115(61%)
	Transport for referred patients	12(6%)
**Reasons for referral (n = 186)**	Lack of blood	55(30%)
	Poor response to treatment	66(36%)
	Lack of IV Fluids	31(17%)
	No beds for admission	16(8.7)
	Others	18(9.6)

*Dangers signs as convulsions or fits, temp above 39.5°C, very pale mucous membranes (anaemia) and unable to localise painful stimuli (coma) Integrated Management of Childhood Illnesses (IMCI).

### Staff and infrastructure

#### Workload capacity

During the survey 18% (131/730) of the recommended Ministry of Health staff positions at the LLHF were filled by qualified personnel. Majority, 86/131 (66%) of care services were performed by nursing assistants. Seventy eight (63%) health workers received written appointment letters and job descriptions at the time of employment to their current positions. Sixteen (12%) health workers were recruited or transferred within 6 months preceding the survey.

#### Supervisory capacity

Support supervision was weak with only 55 (42%) of the health workers supervised on management of malaria in last 6 months. Supervision was irregular and no clear lines of accountability to the immediate supervisors. Whereas all the health workers felt support supervision was useful as an incentive, 43% (56/131) had received on-job training and/or supervision on malaria management in the 12 months preceding this survey. Only 10% (12/131) had been supervised by the person in charge of malaria at the district health office in the foregoing 6 months.

#### Facility capacity

Only 9 (15%) of the larger LLHF had functional in-patient facilities, and these were predominately used by pregnant women. Screening of very sick patients was reported in 112 (85%) of all LLHF but only 33 (26%) put a mark on the patients file (triage) for immediate attention while waiting in the queues

#### Support service capacity

All the smaller LLHF lacked running water and adequate secure storage space for medical supplies including RDTs. Only 30% of the larger LLHF offered laboratory services. All LLHF lacked adequate infection control equipment and mechanisms of safe disposal of biological waste. There were no standardized internal and external quality control systems. The limited quality control measures reported at some facilities included comparing results/slides with negative and positive controls in 7/18 (38%) LLHF, but none of the LLHF had ever participated in any external quality assurance accreditation scheme. Only 3 (17%) LLHF reported regular reagent changing as a means of quality control.

#### Referral practices

All health workers reported use of written referral notes to refer patients to higher level health care facilities. On reviewing charts of 186 patients admitted at the district hospitals following referral LLHF; 143/186 (77%) were children less than 5 years of age, 130 (70%) patients had received pre-referral anti-malarial drugs (oral and intramuscular quinine), but none had received any rectal suppository (rectal artesunate). Referral notes had been provided to 115 (62%) and transport support to 12 (6%) of the patients. The main reasons for referral were poor response to treatment among 66 (36%), need for blood transfusion among 55 (30%) and need for intra-venous fluids among 31(17%) of referred patients. Overall, 10% (18/186) patients had received adequate referral care.

### Structures, systems and roles

#### Systems capacity

All LLHF had registers for patients seen in out-patients departments (OPD) and majority (97%) were up to date. There was no uniform method of recording and storing vital mortality data at the LLHF. Only 25 (20%) of the registers had complete information on malaria mortality. Information and data at LLHF was managed manually and summary totals were transmitted to higher lever facilities and health sub-district for collating and subsequently reported to the Ministry of Health. Communication with higher level health facilities like district hospitals was done using personal mobile telephones except at one facility where radio call communications was mentioned.

#### Structural and role capacity

The LLHF report to the MoH through the health sub-district and district. Over 84% (64/77) of the LLHF held regular staff meetings, 47% held monthly meetings, but proceedings of such meeting were not documented or available for review.

All administrative roles were carried out by the head of the LLHF. Only 15% of the LLHF were led by a health worker with appropriate qualifications (Table 1). The head of facility allocated duty rosters, submitted monthly reports and compiled lists of supplies to be procured through the supervising health sub-district. There were no decision-making forums at LLHF. The staff recruitment and governance of the LLHF is managed centrally (at the district) so it was difficult to assess in this survey.

## Discussion

This study assessed the health system’s capacity to provide parasite-based malaria diagnosis (PMD) at LLHF in preparation for the nation-wide scale-up of RDTs in Uganda. There was limited capacity in terms of 1) diagnostic tools and supplies, 2) personnel skills and knowledge, 3) staff workforce and infrastructure, and 4) structure, systems and roles necessary for successful implementation of malaria diagnosis interventions at LLHF in Uganda.

### Tools for diagnosis and case management

The tools necessary to support clinical practice were limited. There was limited use of microscopy and limited availability of RDTs. Provision of testing tools like RDTs and microscopes is only a starting point of the PMD intervention. Subsequently, routine use of diagnosis then implies health workers’ ability to adopt PMD as a shift from presumptive treatment of malaria. This initial survey considered coverage rather than quality of microscopy. We recommend further studies to evaluate the quality of microscopy given the limited availability of laboratory technologists at the LLHF. In addition to limited diagnostic tools, the quality of malaria case management depends largely on availability of anti-malarial drugs. This survey found that over half of the LLHF faced frequent and prolonged stock-outs of essential medical supplies such as: anti-malarial drugs, syringes and intravenous fluids. Although there is no documented minimum anti-malarial drug stock-out period compatible with adequate malaria case management in an endemic region, the authors felt that drug stock out periods of over three months represent an inadequate stock management system, which is a major drawback to malaria diagnosis and treatment. These levels of stock-outs are similar to those reported in a recent study in two districts in Uganda [13] highlighting the limited health system capacity to manage and maintain a supply chain for routine commodities. This is likely to limit effective implementation of diagnostics such as RDTs that will be provided through the current delivery mechanism for medicines, consumables and reagents. There is need for a well coordinated inventory system at the LLHF in order to prevent stock-outs of anti-malarial drugs and other medical supplies. Given that malaria accounts for 30-50% of out-patient hospital visits and 8% of under-five mortality [14], provision of medical supplies at LLHF is a critical step in reducing infant mortality. Similarly, availability of diagnostic tools is critical to appropriate management of malaria and non-malaria febrile illnesses given that the latter contributes a higher mortality rate among children < five years. Therefore, investment in diagnostic tools should be equally matched with intensive efforts to provide essential medicines and health supplies in order to ensure improved quality of health care delivery. In addition, the procurement capacity should be strengthened to ensure timely delivery and equitable distribution of essential medical supplies at LLHF.

### Skills and knowledge capacity

There was limited health worker capacity in terms of numbers, level of training and technical skills required for quality health care delivery. Only 18% of the recommended staff positions at the LLHF were occupied by qualified personnel. Majority (66%) of the health workers were nursing assistants; a cadre with limited pre-service training. This survey found that the nursing assistants had limited technical skills to enable effective malaria case management. Moreover there was limited support supervision from senior health workers which would otherwise improve the performance of nursing assistants.

Since RDTs require less skill than microscopy [15-19], and provide accurate diagnosis [20]; they can be used to scale up PMD especially at LLHF where microscopy is not feasible due to personnel and facility limitations. This is a potential area for utilization of task-shifting to delegate RDTs to less specialized health workers [21,22]. It is therefore possible to deploy RDTs after brief training of care providers using job aides [9]. Whereas health worker training has been shown to be critical for improving case management, other systems capacity needs must be addressed alongside implementation of RDTs. In addition to guidelines that emphasize PMD before therapy, it is critical to train health workers on the diagnosis and management of alternative causes of fever [23] in order to maximize impact of diagnostics [24]. Given the limited technical skills among the health care providers at LLHF, support supervision and on-job training is required to promote prescription behavior change and maintain high quality health care delivery.

Effective supervision requires qualified health workers to mentor junior staff in a systematic manner as a form of ‘in-service’ training. The job descriptions of junior staff should include clear lines of accountability to their supervisors. However this survey found that support supervision was hampered by lack of qualified staff and logistics. In addition, one third of the health workers did not have appointment letters or job descriptions to determine how they would be supervised. There is evidence that supervision and simple guidelines after deploying RDTs positively impact on prescription behavior [10,25-27] and limited supervision causes sub-optimal utilization of tests kits [28-31]. It is therefore important to re-emphasize the roles of qualified supervisors at LLHF as part of malaria diagnosis intervention and general improvement of the quality of health care.

### Staff and infrastructure capacity

Introducing a new intervention into routine practice should consider its impact on workload or patients’ waiting time at the facility. For example, using RDTs to test all febrile patients prior to treatment may increase health workers’ workload. Therefore health workers require motivation to sustain performance.

We found a shortage of qualified health workforce in all the LLHF. Health workers were already overworked since only 1 out of 5 (20%) of the required positions were filled with qualified health workers. This level of staffing is way below the 80% national Ministry of Health recruitment target. It is likely that this shortage of staff affected the quality of leadership since only 15% of the larger LLHF had appropriately trained leaders. At several LLHF, nurses and nursing assistants provided a broad range of services under minimal supervision due to shortage of qualified health workers. This causes an ethical challenge because nursing assistants are neither recognized nor regulated by any medical professional body in Uganda. Therefore they offer unregulated task-shifting services and it is difficult to monitor their competences, training needs as well as professional discipline. There is need for an institutional and legal framework to protect both the service providers and the patients served by this category of health care providers.

One third of the LLHF had microscopy although they lacked a proper quality control and quality assurance system. There were no defined procedures for monitoring analytical performance, consistent documentation and resolution of quality control (QC) issues. The laboratories were not participating in any external quality assurance scheme. These systems are vital to the maintenance of credible RDT results because inaccurate results have been shown to demoralize health workers, and damage the credibility of PMD [28]. Quality assurance schemes need to be put in place to support RDTs deployment at the peripheral levels. These schemes will ensure that periodic blood smears are prepared and sent to a reference laboratory to validate RDT results using microscopy or other gold standards. In collaboration with WHO/TDR and other agenies, Foundation for Innovative New Diagnostics (FIND) has developed some prototype positive control wells (PCWs) containing antigens that may be used to control the quality of RDTs at the peripheral level. Other centrally planned lot and batch testing will not require local capacity building at LLHF but rather coordination and communication [32,33].

### Structure, systems and roles capacity

Rational referral is part of effective resource utilization. Only one-in-ten of the patients received adequate referral. The concept of a rational and effective referral system was non functional at all LLHF. There were no clear linkages between the various tiers of the health care delivery systems. For effective malaria case management, key elements such as blood transfusion services and oxygen and admission beds were lacking at LLHF. Some of the reasons for referral reported in this study are preventable if the supply chain management is operational without frequent stock-outs of essential supplies like antimalarial drugs and intravenous fluids. Poor response to treatment was one of the four leading causes of referral but this was clearly in absence of confirmatory test or documentation of the effective treatment [34]. Therefore, improving health facilities’ pharmaceutical management capacity is required to ensure non-interrupted supply of pre-referral medications like rectal artesunate as a strategy to improve outcomes of referred patients. It is therefore critical to include inventory management as part of the training package for malaria diagnostics.

Most of the LLHF reported health data to the district through a paper based system. However since 2012, an SMS-based reporting (mTRAC) system is being rolled out to improve local utilization of this data and to report stock-outs of medicines and health supplies. This was not yet in use at the LLHF surveyed. The LLHF leaders need skills to utilize consumption data to make appropriate orders and prevent stock-outs or drug expiry at the facilities. There is need to develop specific capacity to manage stock data at both the LLHF and district level including utilization of simple technology to prevent stock-outs and wastage of resources.

### Limitations

This study did not evaluate governance capacity at the district level which is important for effective service delivery at LLHF. For example, the authority and responsibility to make the decisions essential to effective performance, regarding schedules, budgets, and staff appointments were vested at higher level facilities within the prevalent decentralized health system.

## Conclusion

Primary health care facilities had inadequate human and infrastructural capacity for effective health care delivery including parasite-based malaria diagnosis. The priority capacity building needs identified include training of health workers to improve personal capacity, increasing workforce capacity, strengthening leadership, supervision, supply chain systems and institutional quality assurance/control systems as well as improving patient referral systems. A systematic capacity building approach is required to address all the relevant components of the health system to improve the quality of health care delivery that includes effective parasite-based malaria case management at primary health care facilities.

## Competing interests

All authors (DJK, DN, JPV, BM, RM, HM, JNK, UD, AT and AJ) declare no competing interest.

## Authors' contributions

JA, DK AT, UD, BM and RM contributed to the study design. JA, DK and HM, coordinated data collection, trained the survey staff and supervised the field work. DJK JA, DN and JNK were responsible for data analysis. DK and DN wrote the draft manuscript. JPV provided overall guidance, reviewed and interpreted the findings. All authors reviewed the results, contributed to and approved the manuscript.

## Pre-publication history

The pre-publication history for this paper can be accessed here:

http://www.biomedcentral.com/1471-2458/12/695/prepub

## Supplementary Material

Additional file 1 **Survey tools used to collect data for malaria baseline survey and capacity building needs.** These four tools were used to collect data in the following categories (but not limited):1) Geographic, Historical and Demographic information, 2) Knowledge on severe malaria and its management, 3)Diagnosis and Treatment, 4) Stocks, 5) Patient triage 6) Referral system, 7) Supervision on Malaria Case Management, 8) Roles and Responsibilities 9) Aides Memoir, 10) Death due to severe Malaria 11)Quality of care, 12) Human resources, 13) Records 14) Supplies and Equipment, 15) Communication, 16) Laboratory diagnosis, 17) Supervision on Malaria laboratory. Click here for file

Additional file 2 **Staffing Norms for Primary Health Care Workers 578 at District, City, Municipality and Town Councils.** The table is an 579 extract from page 4-5 of original document by Ministry of Health 580 Uganda- 2006 showing the staffing levels for lower level health facilities 581 Kyabayinze et al. BMC Public Health 2012, 12:695 Page 7 of 9 http://www.biomedcentral.com/1471-2458/12/695 582 (Health centre II and III). The national target is to have 80% of the 583 positions filled. Comprehensive nurses are a those that have both general 584 nursing and midwifery skills. Click here for file
