# Supplementary material for: Parasite-based malaria diagnosis: Are Health Systems in Uganda equipped enough to implement the policy?
Source: BMC Public Health. 2012 Aug 24;12:695. doi: 10.1186/1471-2458-12-695 (PMC3490993; doi:10.1186/1471-2458-12-695)
Supplement: Additional file 1 — Survey tools used to collect data for malaria baseline survey and capacity building needs. These four tools were used to collect data in the following categories (but not limited):1) Geographic, Historical and Demographic information, 2) Knowledge on severe malaria and its management, 3)Diagnosis and Treatment, 4) Stocks, 5) Patient triage 6) Referral system, 7) Supervision on Malaria Case Management, 8) Roles and Responsibilities 9) Aides Memoir, 10) Death due to severe Malaria 11)Quality of care, 12) Human resources, 13) Records 14) Supplies and Equipment, 15) Communication, 16) Laboratory diagnosis, 17) Supervision on Malaria laboratory. [file 1471-2458-12-695-S1.docx]

**Survey tool I : Malaria baseline survey - For Outpatients’ Health Centre II and III**

***Instructions***

*1. Complete the blank space with the answers given*

*2. Select the most appropriate option by clearly ticking the correct one/s with a pencil.*

*3. Do not prompt with the listed answers unless prompting is specified*

*4. If the Health centre has admission facilities then complete the form for Inpatients as well*

**A. Geographic, Historical and Demographic information (GD)**

1. Name of health facility: ________________________________

2. Cadre to be interviewed:

i. Nursing Aid / Asst

ii. Clinical Officer

iii. Nursing Officer

iv. Enrolled Nurse

v. Midwife only

vi. Comprehensive nurse

vii. MO

viii. SMO

ix. Consultant

x. Other_________________________________

3. Duration you have been at current post:

i. < 6 mths

ii. 6 – 12 mths

iii. > 12 mths


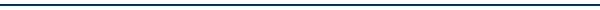


**B. Knowledge on severe malaria and its management (KW)**

1. Ask to list the types of severe malaria *(tick those mentioned without prompting)*

i. Cerebral malaria

ii. Severe anaemia

iii. Renal failure

iv. Pulmonary oedema

v. Hypoglycaemia

vi. Shock

vii. Spontaneous bleeding

viii. Repeated convulsions

ix. Acidosis

x. Haemoglobinuria

2. Commonest type of severe malaria seen in the last week [ ]

3. Ask to list danger signs that indicate the need for urgent attention in a very sick patient *(tick those mentioned)*

i. Rapid breathing [ ]

ii. Deep breathing [ ]

iii. Chest indrawing [ ]

iv. Unable to localise painful stimuli [ ]

v. Extreme generalised body weakness / cannot feed [ ]

vi. Convulsions / fits [ ]

vii. Very pale mucous membranes / palms [ ]

viii. Yellowing of the white part of the eyes [ ]

ix. Body temperature above 39.5^o^C [ ]

x. Has sunken eyes / fontanelle [ ]

xi. Has reduced skin turgor [ ]

xii. Repeated vomiting [ ]

xiii. Does not know any [ ]

4. Which of the following practices are important in saving the lives of patients with severe malaria

| Rate from 1-5 as below |  |
| --- | --- |
| Not important practice for saving life | 1 |
| Important practice for saving life | 2 |
| Very important practice for saving life | 3 |
| No idea / No response | 4 |
| Not applicable | 5 |

i. Take a long and detailed history [ ]

ii. Carry out a short but thorough examination [ ]

iii. Measure and monitor respiratory rate in those under 5 years [ ]

iv. Measure and monitor body temperature [ ]

v. Tepid sponging if febrile [ ]

vi. Fanning if febrile [ ]

vii. Measure blood glucose if unconscious [ ]

viii. Identify patients with danger signs from other patients [ ]

ix. Start prompt treatment with IV quinine [ ]

x. Do blood slide to confirm malaria parasites in blood [ ]

xi. Nurse in the lateral positions if unconscious [ ]

xii. Blood transfusion for those with severe anaemia [ ]

xiii. Educate attendants on bednets before starting treatment [ ]

5. Are there other conditions that can present like severe malaria ( Y / N )

6. If Y, which ones occur in your unit?

i. _________________

ii. ________________

iii. ________________

iv. ________________


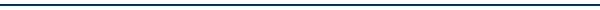


**C. Diagnosis and Treatment (DT)**

1. Do you usually make a final diagnosis based on:

i. clinical features only (presumptive) [ ]

ii. clinical features and diagnostic tests (confirmatory) [ ]

iii. Both [ ]

2. What antimalarial drugs do you routinely give severe malaria cases (as treatment)i. Quinine

ii. Chloroquine

iii. Artemether

iv. Artemether-lumefantrine

v. Amodiaquine

vi. Artesunate

vii. Chloroquine+SP tablets

viii. Other _______________

xi. Referred without treatment

3. What route do you routinely use to give the antimalarial?

i. IM injection

ii. IV infusion

iii. Oral tablets or syrup

iv. Rectal

v. Not applicable

4. Are children weighed before an antimalarial is prescribed? ( Y / N )

5. A patient presents to you with history of fever for 4 days, associated with convulsions and now is unconscious. You think this patient has severe malaria. What antimalarial treatment will you give this patient?

i. Quinine (Y/N)

ii. Artemether (Y/N)

iii. Artesunate (Y/N)

iv. Others, specify______________________________________________

6. For how long will you administer the antimalarial for?_________________

7. If this patient was a 4 year old child, write the exact prescription of the antimalarial you would prescribe

________________________________________________________________

7. If this patient was an adult, write the exact prescription of the antimalarial you would prescribe

_________________________________________________________________

8. What additional supportive treatment would you give to these patients?

i.__________________________________________________________

ii.__________________________________________________________

iii.__________________________________________________________

iv.__________________________________________________________

v.__________________________________________________________

9. What problems do you face in managing severe malaria cases in your unit:

a) ___________________________________________________

b) ___________________________________________________

c) ___________________________________________________

d) ___________________________________________________

10. Have you had the opportunity to improve your skills at severe malaria case management in the last 12 months? ( Y / N )

11. If Y, how?

i. At workshops

ii. Teaching by a colleague/senior from within the health facility

iii. Teaching by someone from outside the health facility

vi. Reading printed material / self teaching

v. Other, specify __________________________

12. Have you ever undergone IMCI training? ( Y / N )


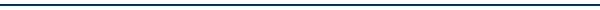


**D. Stock (ST)**

*Complete the checklist for supplies and equipment*

1. How often did you get stock outs lasting more than one week of the items listed below in the previous three months in your unit?

| Code |  |
| --- | --- |
| Not available (but should be) | 0 |
| Available and never out-of-stock | 1 |
| 1-2 stock-outs | 2 |
| 3-4 stock-outs | 3 |
| More than 4 stock-outs | 4 |
| Not applicable | 5 |

| **Items** | **Frequency** | **Main reason for stock-out** |
| --- | --- | --- |
| i. Quinine (parenteral) |  |  |
| ii. Normal saline |  |  |
| iii. 50% dextrose |  |  |
| iv. Blood for transfusion |  |  |
| v. IV giving sets |  |  |
| vi. Blood transfusion set |  |  |
| vi. Syringes |  |  |

2. Are there particular months of the year when you are more likely to get stock-outs of:

i. Quinine inj ( Y / N )

ii. Blood for transfusion ( Y / N )

3. If Y, when? i. Quinine inj ___________________________________

ii. Blood _______________________________________


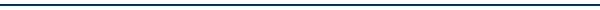


**E. Patient triage (PT)**

1. Who is usually the first to meet the patient and attendants when they arrive at the health facility?

i. 9am - Midday: _________________________________________

ii. 10pm – 1am: _________________________________________

2. Is there a method of screening very sick patients from the queue?

( Y / N )

3. If Y, who identifies them? _______________________________

______________________________________________________

4. If Y, what are the most useful signs that are used to identify very sick patients in the queue?

i. ________________________________________________

ii. _______________________________________________

iii. _______________________________________________

5. If Y, are very sick patients marked in any way? ( Y / N )

6. If Y, how are they marked? ________________________________

7. If N, how would you want them marked? ___________________

_______________________________________________________

8. What is done for those who are screened? ___________________

________________________________________________________


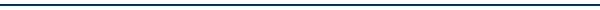


**F Timing (TI)**

1. Complete the table below using information from the health worker

| Code: |  |
| --- | --- |
| Within 30 mins | 1 |
| >30 mins – 1 hour | 2 |
| >1 hour – 3 hours | 3 |
| >3 hours | 4 |
| Not applicable | 5 |

| **Component of triage** | **9am - midday** | **10pm – 1am** |
| --- | --- | --- |
| i. Arrival to seeing the relevant health worker |  |  |
| ii. Clinical assessment to getting results of blood smear |  |  |
| iii. Clinical assessment to getting first treatment dose |  |  |
| v. Clinical assessment to getting a blood transfusion |  |  |
| vi. Getting referral note to departure from the health facility |  |  |


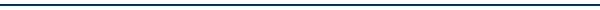


**G. Referral system (R)**

1. Number of patients with severe malaria that have been referred that day [ | ]

2. What are the reasons why you decide to refer patients with severe malaria?

i. Lack of blood for transfusion at the facility (Y / N)

ii. Poor response to treatment given (Y / N)

iii. Lack of I.V fluids (Y / N)

iv. Lack of Oxygen (Y / N)

v. No beds available to admit patient (Y / N)

vi. Others, specify___________________________________________

3. Do you use the presence of some clinical signs to make referral decisions?

(Y / N)

4. If Y, what signs do you use?

i. Rapid breathing [ ]

ii. Deep breathing [ ]

iii. Chest indrawing [ ]

iv. Unable to localise painful stimuli [ ]

v. Extreme generalised body weakness / cannot feed [ ]

vi. Convulsions / fits [ ]

vii. Very pale mucous membranes / palms [ ]

viii. Yellowing of the white part of the eyes [ ]

ix. Body temperature above 39.5^o^C [ ]

x. Has sunken eyes / fontanelle [ ]

xi. Repeated vomiting [ ]

xii. Others, specify________________________________________

5. When you refer to another health facility do you give any pre-referral medications ( Y / N )

6. If Y, what do you give? ___________________________________

________________________________________________________

7. If N, why not? __________________________________________

8. Do you give a referral note? ( Y / N )

9. Where do you refer the patients to (name)?

i. _______________________ approx distance from unit ________ km

ii. _______________________ approx distance from unit ________ km

10. Do you give the attendants directions to get to the health facility? ( Y / N )

11. Do you tell the attendants what form of transport to use? (Y / N )

12. Do you tell them where to report when they get to the health facility? ( Y / N )

13. Do you give any other advice ( Y / N )

11. If Y, what? ____________________________________________

14. Do you have a method of finding out the outcome of the referral? ( Y / N )

15. If Y, how _____________________________________________

16. If N, would you like to know the outcome? ( Y / N )


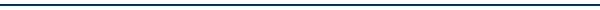


**H. Supervision on Malaria Case Management (SU)**

1. Have you undergone any form of supervision on the management of malaria in the last 6 months? ( Y / N )

2. If Y, were you comfortable with the process? ( Y / N )

3. Who has supervised you in the last 6 months?

Within the health facility

i. Colleague [ ]

ii. Immediate senior [ ]

iii. Head of unit [ ]

iv. Head of health facility [ ]

From outside the health facility

v. Malaria focal person [ ]

vi. Malaria zonal coordinator [ ]

vii. Staff from health subdistrict [ ]

viii. Consultant from the nearest referral hospital [ ]

ix. Ministry of Health technical staff [ ]

x. Health worker from abroad [ ]

4. How often have you been supervised in the last 6 months?

i. Once

ii. Twice

iii. Thrice

iv. Monthly

v. None

5. What methods have you been supervised with in the last 6 months?

i. Direct observation of care

ii. Interviews

iii. Inspection

iv. Feedback

v. Problem-solving

vi. Coaching

vii. Training

viii. Decision-making

ix. Clinical audit

x. Other, Specify_________________

6. Do you feel support supervision for malaria is useful? ( Y / N )

7. If yes, how is it useful?

i. Improved competence / skills

ii. Improved compliance with national guidelines

iii. Improved care given to patients

iv. Improved motivation

v. Other, specify _____________________________________

8. Can you list any international organisations or NGOs that are involved in malaria work where your facility is located?

1. _________________________________________
2. _________________________________________
3. _________________________________________

9. How have these international organisations or NGOs been useful to you or the community?

i._________________________________________

ii._________________________________________

iii._________________________________________

iv._________________________________________


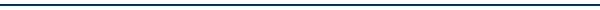


**I. Roles and Responsibilities (RR)**

1. Were you given a job description when you started your current post? ( Y / N )

2. If Yes, was it i. written or ii. verbal

3. What would you consider as your role in the routine management of patients with severe malaria?

i. _______________________________________________________

ii. ______________________________________________________

iii. ______________________________________________________


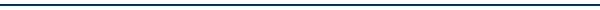


**J. Aides Memoir (AM)**

1. Which of the following severe malaria case management aides are available at the unit?

i. Posters on the wall ( Y / N )

ii. Wall charts ( Y / N )

ii. Leaflets / Pamphlets ( Y / N )

iii. Reference textbooks ( Y / N )

iv. Desk aids ( Y / N )

Others, specify _________________________________________

2. Which do you prefer as a reminder?

| Rate from 1 to 5 as below |  |
| --- | --- |
| Not useful | 1 |
| A good reminder | 2 |
| A very good reminder | 3 |
| No idea / No response | 4 |
| Not applicable | 5 |

i. Posters on the wall [ ]

ii. Wall charts [ ]

ii. Leaflets / Pamphlets [ ]

iii. Reference textbooks [ ]

iv. Desk aids [ ]

Others, specify _________________________________________


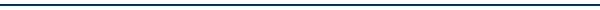


**K. Adverse reactions (AR)**

1. Do you inform attendants of the adverse reactions of the antimalarial which the patient is getting? ( Y / N )

2. If Y, which ones do you mention for quinine

Drug Adverse Reaction

Quinine i. ________________________________

ii. ________________________________

iii.________________________________

3. If N, why not_________________________________________

4. Do you record and report suspected adverse reactions of any of the drugs that you use in your facility? ( Y / N )

5. If Y, were do you record _______________________________________

6. Who do you report to __________________________________________

7. If N, why not? __________________________________________


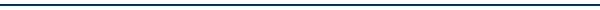


**L. Death due to severe Malaria**

3. On what days of the week do most of these deaths occur?

i. Monday to Wednesday ( Y / N )

ii. Thursday to Friday ( Y / N )

iii. Weekends ( Y / N )

4. At what times do these deaths commonly occur?

i. Mornings (Y/N)

ii. Afternoons (Y/N)

iii. Evenings (Y/N)

iv. Nights (Y/N)


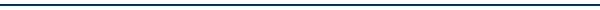


**M. Quality of care (QC)**

1. How do you rate the quality of care that your unit gives to patients with severe malaria? [ ]

| Rate from 1 to 5 as below |  |
| --- | --- |
| Poor quality | 1 |
| Good quality | 2 |
| Very good quality | 3 |
| No idea / No response | 4 |
| Not applicable | 5 |

i. Quality of diagnosis [ ]

ii. Quality of treatment [ ]

iii. Quality of nursing care [ ]

iv. Quality of supportive care [ ]

v. Quality of follow-up [ ]

vi. Quality of management of the health facility [ ]

2. What specific aspects of care are weak in your health facility?

i. ________________________________________________________

ii. ________________________________________________________

iii. _______________________________________________________

3. What specific aspects of care are done very well in your health facility?

i. ________________________________________________________

ii. ________________________________________________________

iii. _______________________________________________________

4. What suggestions do you have to improve the quality of care given to patients with severe malaria in your health facility?

i. __________________________________________________________

ii. __________________________________________________________

iii. _________________________________________________________

iv. _________________________________________________________

Date: ___ / ____ / 2009 Time _______ am /pm

Completed by: _________________ (name)

**Survey tool II: Checklist for each Health Facility**

***Instructions***

*1. Complete the blank spaces*

*2. Select the most appropriate option by clearly ticking the correct one/s with a pencil.*

*3. Complete this checklist by observing what goes on in the units of the health facility*

**A. Geographic, Historical and Demographic information (GDC)**

1. Name of health facility: ________________________________

2. Grade of health facility

i. HC II

ii. HC III

iii. HC IV

iv. District Hospital

v. Other ______________

3. Type of health facility

i. Government facility

ii. Faith-based facility

iii. Private-for-profit based facility

iv. Other ___________________

4. Name of village: _________________ 5. Parish: __________________

6. Subcounty: _____________________ 7. District: __________________

8. Approximate size of population in catchment area __________________


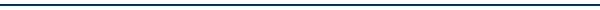


**B. HUMAN RESOURCES (HRS)**

Record the number of personnel by cadre, carefully recording the following information:

- Number of staff employed in the facility
- Number of staff scheduled to be on duty on the day of survey
- Number of staff present during the survey

| **Cadre** | **Number required according to MOH staffing norms** | **Number of staff employed in facility** | **Number of staff scheduled for duty today** | **Number of staff present on duty today** |
| --- | --- | --- | --- | --- |
| Medical Doctor |  |  |  |  |
| Health officer |  |  |  |  |
| Clinical Nurse |  |  |  |  |
| Public Health Nurse |  |  |  |  |
| Midwife |  |  |  |  |
| Comprehensive Nurse |  |  |  |  |
| Community health worker |  |  |  |  |
| Nursing Aids |  |  |  |  |
| Laboratory technicians |  |  |  |  |
| Nursing Aids |  |  |  |  |
| Other (Specify) |  |  |  |  |


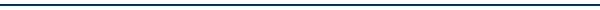


**C. Records (RCC)**

1. Is there a register for keeping record of patients seen in OPD ( Y / N )

2. If Y, is it uptodate (by yesterday) ( Y / N )

3. Is there a register for keeping record of patients admitted ( Y / N / NA)

4. If Y, is it uptodate (by yesterday) ( Y / N )

5. Do the records note:

i. Age of patient ( Y / N )

ii. Type of severe malaria manifestation ( Y / N )

iii. If microscopy was performed ( Y / N )

iii. Records are not clear

6. In April 2009, what was the number of severe malaria cases?

i. Referred _________________ or Not applicable

ii. Admitted _________________ or Not applicable

iii. Died in health facility _______________ or Not applicable

8. Of the patients admitted with severe malaria last year, how many died? (Also mention the total number admitted with severe malaria during this period)

i. Number of adults__________________________________

ii. Number of children________________________________

9. Of the patients admitted with severe malaria last month, how many died? (Also mention the total number admitted with severe malaria during this period)

i. Number of adults__________________________________

ii. Number of children________________________________

10. What are the common causes of death in patients presenting with severe malaria at this health facility

1. Cerebral malaria (Y/N)

2. Severe anaemia (Y/N)

3. Hypoglycaemia (Y/N)

4. Severe dehydration (Y/N)

5. Respiratory distress (Y/N)

6. Others, specify________________________________

11. How are records of death kept in this facility?

1. Inpatient register

2. Death register

3. Other, specify____________________________________

12. Were source documents of death records verified by the interviewer? (Y/N)

13. If yes, comment on the quality of records

1. Good quality

2. Poor quality

3. Accurate

4. Inaccurate/incomplete

5. Other, specify__________________________________________________


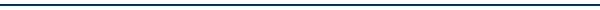


**D. Supplies and Equipment (SAEC)**

1. Which of the following diagnostic facilities are available and functional **within** the unit specified *(A=available, F=functional, AF=available and functional, N=None, use these letters to indicate the pertaining situation)*

| **Test** | **OPD** | **Children ward** |
| --- | --- | --- |
| i. No diagnostic facilities |  |  |
| ii. Malaria Rapid test kit |  |  |
| iii. Parasight F |  |  |
| iv. ParaCheck |  |  |
| v. Optimal |  |  |
| vi. Hand-held Glucometer |  |  |
| vii. Glucose dipstick |  |  |
| viii. Urine dipstick |  |  |
| ix. Hb colou  r scale |  |  |
| x. HemoCue™ haemoglobinometer |  |  |
| xi. Microscopy |  |  |

2. Complete this table for the OPD noting the supplies that the staff in the OPD currently have **access to**.

|  | **Item** | **Specification** | **√/×** | **NA** |
| --- | --- | --- | --- | --- |
|  | **Drugs** |  |  |  |
| 1 | Quinine | Injectable |  |  |
| 2 |  | Oral |  |  |
| 3 | Chloroquine | Injectable |  |  |
| 4 | Sulphadoxine-pyrimethamine | Oral |  |  |
| 5 | Artemether-lumefantrine | Oral |  |  |
| 6 | Artemether | Injectable |  |  |
| 7 | Artemisinin | Rectal |  |  |
| 8 | Artesunate | Iv |  |  |
| 9 |  | Rectal |  |  |
| 10 | Arteether | Injectable |  |  |
| 11 | Diazepam | Injectable |  |  |
| 12 |  | Rectal |  |  |
| 13 | Dextrose | 50% |  |  |
| 14 |  | 30% |  |  |
| 15 |  | 25% |  |  |
| 16 | Paracetamol | Oral |  |  |
| 17 |  | suppositories |  |  |
| 18 | Phenobarbitone | Injection |  |  |
| 19 | Furosemide | Injection |  |  |
|  | **Item** | **Specification** | **√/×** | **NA** |
|  | **Fluids** |  |  |  |
| 20 | Dextrose | 5% |  |  |
| 21 |  | 10% |  |  |
| 22 |  | 50% |  |  |
| 23 | Saline | 0.9% |  |  |
| 24 | Darrow’s solution | Half strength |  |  |
| 25 |  | Full strength |  |  |
| 26 | Ringer lactate | 500ml |  |  |
| 27 | Fluid bottles | 100ml |  |  |
| 28 |  | 200ml |  |  |
| 29 |  | 500ml |  |  |
| 30 | Water for injection |  |  |  |
|  | **Medical** |  |  |  |
| 31 | NG tube | Paediatric sizes |  |  |
| 32 |  | Adult sizes |  |  |
| 33 | IV giving sets |  |  |  |
| 34 | Blood transfusion sets |  |  |  |
| 35 | IV cannulae | Paediatric sizes |  |  |
| 36 |  | Adult sizes |  |  |
| 37 | Scalp vein butterfly needles |  |  |  |
| 38 | Needles disposable |  |  |  |
| 39 | Syringes | 2ml |  |  |
| 40 |  | 5ml |  |  |
| 41 |  | 10ml |  |  |
| 42 |  | 20ml |  |  |
| 43 | Syringe feeding | 50/60ml |  |  |
| 44 | Gloves | Sterile |  |  |
| 45 |  | Disposable |  |  |
| 46 | Cotton wool |  |  |  |
| 47 | Adhesive tape |  |  |  |
| 48 | Lancets |  |  |  |
| 49 | Oxygen in cylinders |  |  |  |
|  | **Equipment** |  |  |  |
| 50 | Thermometer |  |  |  |
| 51 | Weighing scale | Hanging/Salter |  |  |
| 52 |  | Electronic |  |  |
| 53 |  | Bathroom |  |  |
| 54 |  | Other |  |  |
| 55 | Examination table |  |  |  |
| 56 | Stethoscope |  |  |  |
| 57 | Clock/Watch |  |  |  |
| 58 | BP machine |  |  |  |
| 59 | Ophthalmoscope |  |  |  |
| 60 | Otoscope |  |  |  |
| 61 | Oral airways |  |  |  |
| 62 | Ambubag |  |  |  |
|  | **Item** | **Specification** | **√/×** | **NA** |
| 63 | Torch |  |  |  |
| 64 | Glucometer |  |  |  |
| 65 | Glucose dipsticks |  |  |  |
| 66 | Urine dipsticks |  |  |  |


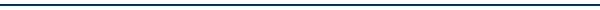


**E. Patient triage (PTC)**

1. Is there a defined triage system in place *(observation)*? ( Y / N )

2. If N, why not? __________________________________________

________________________________________________________

3. What is the entry point to the facility ___________________________________

4. Visible directions tell people where to go ( Y / N )

5. Screening of sick patients at OPD queue ( Y / N / NA )

6. Separate lines for children and adults at OPD queue ( Y / N / NA )

7. Screening of sick patients for urgent treatment on admission queue ( Y / N / NA )

8. Urgent attention given to sick patients at OPD queue ( Y / N / NA )

9. Urgent attention given to sick patients on admission queue ( Y / N / NA )

10. Lab requests marked for urgent response ( Y / N / NA )

11. Lab results that are urgent are given priority ( Y / N / NA )

12. Lab results that are urgent are returned to requester as priority ( Y / N / NA )

13. Describe any other features not captured above _________________________

___________________________________________________________________


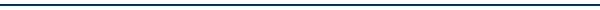


**F. Aides Memoir (AMC)**

1. Which of the following severe malaria case management aides are located in **visible** areas for the staff in the **OPD**?

i. Posters on the wall ( Y / N )

ii. Wall charts ( Y / N )

ii. Leaflets / Pamphlets ( Y / N )

iii. Reference textbooks ( Y / N )

iv. Desk aids ( Y / N )

Others, specify _________________________________________

2. Which of the following severe malaria case management aides are located in **visible** areas for the staff at the **children’s ward**?

i. Posters on the wall ( Y / N )

ii. Wall charts ( Y / N )

ii. Leaflets / Pamphlets ( Y / N )

iii. Reference textbooks ( Y / N )

iv. Desk aids ( Y / N )

Others, specify _________________________________________


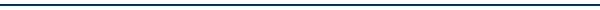


**G. Communication (COC)**

1. Is there an easy and quick means of communicating with the other departments within the health facility ( Y / N )

2. If Y, which forms of communication exist

i. Direct communication in a small unit [ ]

ii. Telephone [ ]

iii. Other_____________________________

3. Is there a means of communicating with the other health facilities in the district?

( Y / N )

4. If Y, which forms of communication exist

i. Radio [ ]

ii. Telephone [ ]

iii. Other_____________________________

5. Are there regular meetings with other staff in the facility? ( Y / N )

6. If Y, how often? ___________________________


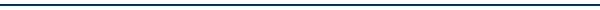


**H. Quality of care (QCC)**

1. How do you rate the quality of care that the unit gives to patients with severe malaria? [ ]

| Rate from 1 to 5 as below |  |
| --- | --- |
| Poor quality | 1 |
| Good quality | 2 |
| Very good quality | 3 |
| No idea / No response | 4 |
| Not applicable | 5 |

i. Quality of diagnosis [ ]

ii. Quality of treatment [ ]

iii. Quality of nursing care [ ]

iv. Quality of supportive care [ ]

v. Quality of follow-up [ ]

vi. Quality of management of the health facility [ ]

2. What specific aspects of care are weak in the health facility?

i. ________________________________________________________

ii. ________________________________________________________

iii. _______________________________________________________

3. What specific aspects of care are done very well in the health facility?

i. ________________________________________________________

ii. ________________________________________________________

iii. _______________________________________________________

Date: ___ / ____ / 2009 Time _______ am /pm

Completed by: _________________ (name)

# Survey Tool III: For pharmacy

***Instructions***

*1. Complete the blank space with the answers given*

*2. Select the most appropriate option by clearly ticking the correct one/s with a pencil.*

*3. Do not prompt with the listed answers unless prompting is specified*

**A. Geographic, Historical and Demographic information (GHD)**

1. Name of health facility: ________________________________

2. Cadre to be interviewed: ______________________________________

3. Duration you have been at current post:

i. < 6 mths ii. 6 – 12 mths iii. > 12 mths

4. Any previous history of training on supply chain management of drugs ( Y / N )


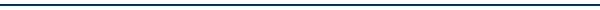


**B. Records (RC)**

1. Register for keeping record of antimalarial usage ( Y / N )

2. If Y, are they uptodate (yesterday) ( Y / N )


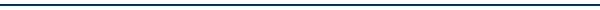


**C. Stock (SK)**

*Complete the checklist for supplies and equipment*

1. How often did you get stock outs lasting more than one week of the items listed below in the previous three months in your unit?

| Code |  |
| --- | --- |
| Not available (but should be) | 0 |
| Available and never out-of-stock | 1 |
| 1-2 stock-outs | 2 |
| 3-4 stock-outs | 3 |
| More than 4 stock-outs | 4 |
| Not applicable | 5 |

| **Items** | **Frequency** | **Main reason for stock-out** |
| --- | --- | --- |
| i. Quinine (parenteral) |  |  |
| ii. Normal saline |  |  |
| iii. 50% dextrose |  |  |
| iv. 5% dextrose |  |  |
| v. Blood for transfusion |  |  |
| vi. IV giving sets |  |  |
| vii. Blood transfusion set |  |  |
| viii. Syringes |  |  |
| ix. Quinine tablets |  |  |

2. Are there particular months of the year when you are more likely to get stock-outs of:

i. Quinine inj ( Y / N )

ii. Blood for transfusion ( Y / N )

iii. Quinine tablets ( Y / N )

3. If Y, when? i. Quinine inj ___________________________________

ii. Blood _______________________________________

iii. Quinine tablets _______________________________


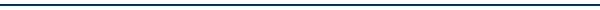


**D. Supplies and Supply management (SSM)**

1. Which of the following are available in the pharmacy unit?

| **Item** | **Specification** | **Yes** | **No** |
| --- | --- | --- | --- |
| **Drugs** |  |  |  |
| Quinine | Injectable |  |  |
|  | Oral |  |  |
| Chloroquine | Injectable |  |  |
| Sulphadoxine-pyrimethamine | Oral |  |  |
| Artemether-lumefantrine | Oral |  |  |
| Artemether | Injectable |  |  |
| Artemisinin | Rectal |  |  |
| Artesunate | IV |  |  |
|  | Rectal |  |  |
| Arteether | Injectable |  |  |
| Diazepam | Injectable |  |  |
|  | Rectal |  |  |
| Paracetamol | Oral |  |  |
|  | Suppositories |  |  |
| Phenobarbitone | Injection |  |  |
| Furosemide | Injection |  |  |
| **Fluids** |  |  |  |
| Dextrose | 50% |  |  |
|  | 30% |  |  |
|  | 25% |  |  |
|  | 10% |  |  |
|  | 5% |  |  |
| Saline | 0.9% |  |  |
| Fluid bottles | 100ml |  |  |
|  | 200ml |  |  |
|  | 500ml |  |  |
| Darrow’s solution | Half strength |  |  |
| **Item** | **Specification** | **Yes** | **No** |
|  | Full strength |  |  |
| Ringer lactate |  |  |  |
| Water for injection |  |  |  |

2. Is there a method in place for preventing stocks-outs? ( Y / N )

3. If Y, what is done? _____________________________________

________________________________________________________

4. If N, why not? __________________________________________

________________________________________________________

5. In the last year has quinine expired in the pharmacy/store? ( Y / N )

6. If Y, why? ________________________________________

7. Is there a method in place for quantifying the antimalarial needs? ( Y / N )

8. If Y, what is it? ________________________________________

________________________________________________________

9. If N, why not? _________________________________________

10. Is your supply of antimalarials sufficient for the patients that are admitted? ( Y / N )

11. If N, which ones are not? ______________________________

12. If Y, how do you ensure adequate supply? _________________

______________________________________________________

13. Do you supply IV fluids in smaller bottles (100ml/200ml) for children ( Y / N )

14. If Y, what have been the benefits _________________________

_______________________________________________________

15. If N, why not? _________________________________________

________________________________________________________

16. Do you keep oxygen for use on the inpatient medical and paediatric wards ( Y / N )

17. If N, why not? ________________________________________

17. What specific aspects of drug management and supply are weak in your health facility?

i. ________________________________________________________

ii. ________________________________________________________

iii. _______________________________________________________

18. What specific aspects of drug management and supply do you think are performed very well in your health facility?

i. ________________________________________________________

ii. ________________________________________________________

iii. _______________________________________________________

19. What suggestions do you have to improve the quality of treatment given to patients with severe malaria in your health facility?

i. __________________________________________________________

ii. __________________________________________________________

iii. _________________________________________________________

iv. _________________________________________________________


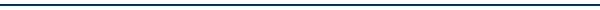


**E. Supervision (SUp)**

1. Have you undergone any form of supervision in the last six months? ( Y / N )

2. If Y, were you comfortable with the process? ( Y / N )

3. Who has supervised you in the last six months?

Within the health facility

i. Colleague

ii. Immediate senior

iii. Head of unit

iv. Head of health facility

From outside the health facility

Specify _________________________

4. How often have you been supervised in the last six months?

i. Once

ii. Twice

iii. Thrice

iv. Monthly

v. None


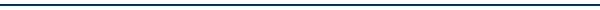


**F. Pharmacovigilance (PV)**

1. Are you informed of any adverse reactions of the drugs that are used in your facility? ( Y / N )

2. If Y, which drugs do you routinely keep records for their reactions? ________________________________________________________

________________________________________________________

3. If Y, who informs you and how? ____________________________

7. If Y, who do you report them to? ___________________________

________________________________________________________

8. If N, why not? __________________________________________

Date: ___ / ____ / 2009 Time _______ am /pm

Completed by: _________________ (name)

# Survey tool V: For laboratory

***Instructions***

*1. Complete the blank space with the answers given*

*2. Select the most appropriate option by clearly ticking the correct one/s with a pencil.*

*3. Do not prompt with the listed answers unless prompting is specified*

**A. Geographic, Historical and Demographic information (GHD)**

1. Name of health facility: ________________________________

2. Cadre to be interviewed:

i. Laboratory assistant

ii. Lab technician

iii. Lab technologist

iv. Senior lab technologist

v. Microscopist

vi. Other _______________

3. Duration you have been at current post:

i. < 6 mths ii. 6 – 12 mths iii. > 12 mths

4. Any training on malaria laboratory diagnosis in the last 12 months ( Y / N )


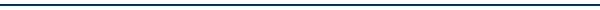


**B. Records (RC)**

1. Register for keeping record of patients investigated ( Y / N )

2. If Y, are they uptodate (yesterday) ( Y / N )

4. Do the records note the:

i. Age of patient ( Y / N )

ii. Type of severe malaria manifestation ( Y / N )

iii. Records are not clear


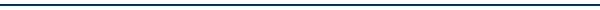


**C. Laboratory diagnosis (LD)**

1. Which of the following diagnostic investigations are done in your laboratory?

| **Investigation** | **( √ ∕ × )** |
| --- | --- |
| Blood smear thick film |  |
| Blood smear thin film |  |
| Blood smear parasite count |  |
| Malaria Rapid test kit |  |
| Hb estimation – Sahl’s method |  |
| Hb estimation – Hb colour scale |  |
| HB estimation - HemoCue™ haemoglobinometer |  |
| Glucose blood concentration – Portable glucometer |  |
| Glucose blood concentration – Glucose-oxidase method |  |
| Glucose blood concentration – Glucostick method |  |
| White blood cell count – total |  |
| White blood cell count – differential |  |
| Red blood cell count |  |
| Platelet count |  |
| Haematocrit |  |
| Blood film |  |
| ESR |  |
| CSF analysis – glucose concentration |  |
| CSF analysis – protein concentration |  |
| CSF analysis – white cell count |  |
| CSF analysis – Gram stain |  |
| CSF analysis – India ink |  |
| CSF analysis – ZN stain |  |
| CSF analysis – culture and sensitivity |  |
| Urinalysis – dipstick |  |
| Serum electrolytes |  |
| Sickling test |  |
| HIV serology |  |
|  |  |

2. Are there any other diagnostic investigations not listed above? ( Y / N )

3. If Y, which ones? _______________________________________

4. In patients with malaria admitted to the wards, are you **routinely** asked to repeat the blood smear for malaria parasites to monitor parasite clearance?

( Y / N )

5. If Y, how often __________________________________________

6. If N, why not? __________________________________________

7. Who **routinely** brings the blood specimens to the laboratory *(how are the blood specimens collected)*? ___________________________________________

8. Are urgent laboratory requests marked in any **special** way? ( Y / N )

9. If Y, in what way? _______________________________________

10. If Y, does the laboratory process them **urgently**? ( Y / N )

11. If Y, what is the average time to get urgent results back to the ward? ______ hrs _______ mins

12. If N, why not _____________________________________

13. Do you have a designated area in the lab where urgent specimens are put? ( Y / N )

14. Where are the results put once investigations have been done?

i. Non-urgent results ______________________________________

ii. Urgent results _________________________________________

15. How do the results get back to the wards? _______________________

16. Do you have a **working** microscopy? ( Y / N )

17. If Y, is it i. mono-ocular or ii. bi-ocular

18. If Y, do you use i. electricity ii. sunlight or iii. both

19. If Y, what quality control measures do you have to make sure that

the results of microscopy are accurate and up to standard on a **regular**

basis? i. __________________________________________

ii. __________________________________________

iii. __________________________________________

20. Do you have the opportunity to discuss your laboratory results with the clinicians on the wards? ( Y / N / NA )


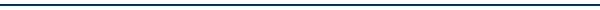


**D. Supervision on Malaria laboratory diagnosis (SUL)**

1. Have you undergone any form of supervision on malaria laboratory diagnosis in the last six months? ( Y / N )

2. If Y, were you comfortable with the process? ( Y / N )

3. Who has supervised you in the last six months?

Within the health facility

i. Colleague

ii. Immediate senior

iii. Head of unit

iv. Head of health facility

From outside the health facility

v. Malaria focal person

vi. Laboratory focal person

vii. Staff from health sub-district

vii. Consultant from the nearest referral hospital

viii. Ministry of Health technical staff

4. How often have you been supervised in the last six months?

i. Once

ii. Twice

iii. Thrice

iv. Monthly

v. None

5. What methods have you been supervised with in the last six months?

i. Direct observation of slides (practical)

ii. Interviews

iii. Inspection

iv. Feedback

v. Problem-solving

vi. Coaching

vii. Training

viii. Decision-making

ix. Clinical audit

x. Other, specify ______________

__________________________

6. Do you feel support supervision for malaria is useful? ( Y / N )

7. If yes, how is it useful?

i. Improved competence / skills

ii. Improved compliance with national guidelines

iii. Improved effectiveness of care

iv. Improved motivation

v. Other, specify _____________________________________


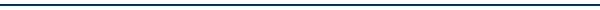


**E. Aides Memoir (AM)**

1. Which of the following malaria laboratory diagnosis aides are **available** at the unit?

i. Posters on the wall ( Y / N )

ii. Wall charts ( Y / N )

ii. Leaflets / Pamphlets ( Y / N )

iii. Reference textbooks ( Y / N )

iv. Desk aids ( Y / N )

Others, specify _________________________________________

2. Which do you prefer as a reminder?

| Rate from 1 to 5 as below |  |
| --- | --- |
| Not useful | 1 |
| A good reminder | 2 |
| A very good reminder | 3 |
| No idea / No response | 4 |
| Not applicable | 5 |

i. Posters on the wall [ ]

ii. Wall charts [ ]

ii. Leaflets / Pamphlets [ ]

iii. Reference textbooks [ ]

iv. Desk aids [ ]

Others, specify _________________________________________

Date: ___ / ____ / 2009 Time _______ am /pm

Completed by: _________________ (name)
