# Supplementary material for: Parasite-based malaria diagnosis: Are Health Systems in Uganda equipped enough to implement the policy?
Source: BMC Public Health. 2012 Aug 24;12:695. doi: 10.1186/1471-2458-12-695 (PMC3490993; doi:10.1186/1471-2458-12-695)
Supplement: Additional file 2 — Staffing Norms for Primary Health Care Workers 578 at District, City, Municipality and Town Councils. The table is an 579 extract from page 4-5 of original document by Ministry of Health 580 Uganda- 2006 showing the staffing levels for lower level health facilities 581 Kyabayinze et al. BMC Public Health 2012, 12:695 Page 7 of 9 http://www.biomedcentral.com/1471-2458/12/695 582 (Health centre II and III). The national target is to have 80% of the 583 positions filled. Comprehensive nurses are a those that have both general 584 nursing and midwifery skills. [file 1471-2458-12-695-S2.docx]

**Additional file 1: Staffing Norms for Primary Health Care Workers at District, City, Municipality and Town Councils (*Page 4-5 of original document* *by Ministry of Health Uganda*) 2006**

|  | **DISTRICT LEVEL** |  | **HCIII** | **HCII** |
| --- | --- | --- | --- | --- |
|  | **Department of district health services** | **SCALE** | **(n).** | **(n)** |
| **1** | **Allied Health Professional** |  |  |  |
|  | Senior Clinical Officer | U4 | 1 | 0 |
|  | Clinical Officer | U5 | 1 | 0 |
|  | Laboratory Technician | U5 | 1 | 0 |
|  | Laboratory Assistant | U7 | 1 | 0 |
|  | Health Assistant | U7 | 1 | 1 |
|  | **Sub-Total** |  | **5** | **1** |
| **2** | **Nursing** |  |  |  |
|  | Nursing Officer (Nursing) | U5 | 1 | 0 |
|  | Enrolled Midwife | U7 | 2 | 1 |
|  | Enrolled Nurse | U7 | 3 | 1 |
|  | Nursing Assistant | U8 | 3 | 2 |
|  | **Sub-Total** |  | **9** | **4** |
| **3** | **Administrative and other staff** |  |  |  |
|  | Health Information Assistant | U7 | 1 | 0 |
|  | Askari (security guard) | U8 | 2 | 2 |
|  | Porter ( casual labourer) | U8 | 2 | 2 |
|  | **sub-Total** |  | **5** | **4** |
|  | **Grand Total** |  | **19** | **9** |

Ref: **Staffing Norms for Primary Health Care Workers at District, City, Municipality and Town Councils MoH Uganda**
